# DDIT: An Online Predictor for Multiple Clinical Phenotypic Drug-Disease Associations

**DOI:** 10.3389/fphar.2021.772026

**Published:** 2022-01-19

**Authors:** Lu Lu, Jiale Qin, Jiandong Chen, Hao Wu, Qiang Zhao, Satoru Miyano, Yaozhong Zhang, Hua Yu, Chen Li

**Affiliations:** ^1^ Department of Human Genetics, Department of Ultrasound and Women’s Hospital, Zhejiang University School of Medicine, Hangzhou, China; ^2^ Zhejiang Provincial Key Laboratory of Precision Diagnosis and Therapy for Major Gynecological Diseases, Hangzhou, China; ^3^ School of Public Health, Undergraduate School of Zhejiang University, Hangzhou, China; ^4^ M&D Data Science Center, Tokyo Medical and Dental University, Tokyo, Japan; ^5^ The Institute of Medical Science, the University of Tokyo, Tokyo, Japan; ^6^ Department of Basic Medical Sciences, Zhejiang University School of Medicine, Hangzhou, China

**Keywords:** drug repositioning, restricted Boltzmann machine, phenotypic types of drug-disease associations, machine learning, indication, side effect, contraindication

## Abstract

**Background:** Drug repurposing provides an effective method for high-speed, low-risk drug development. Clinical phenotype-based screening exceeded target-based approaches in discovering first-in-class small-molecule drugs. However, most of these approaches predict only binary phenotypic associations between drugs and diseases; the types of drug and diseases have not been well exploited. Principally, the clinical phenotypes of a known drug can be divided into indications (Is), side effects (SEs), and contraindications (CIs). Incorporating these different clinical phenotypes of drug–disease associations (DDAs) can improve the prediction accuracy of the DDAs.

**Methods:** We develop Drug Disease Interaction Type (DDIT), a user-friendly online predictor that supports drug repositioning by submitting known Is, SEs, and CIs for a target drug of interest. The dataset for Is, SEs, and CIs was extracted from PREDICT, SIDER, and MED-RT, respectively. To unify the names of the drugs and diseases, we mapped their names to the Unified Medical Language System (UMLS) ontology using Rest API. We then integrated multiple clinical phenotypes into a conditional restricted Boltzmann machine (RBM) enabling the identification of different phenotypes of drug–disease associations, including the prediction of as yet unknown DDAs in the input.

**Results:** By 10-fold cross-validation, we demonstrate that DDIT can effectively capture the latent features of the drug–disease association network and represents over 0.217 and over 0.072 improvement in AUC and AUPR, respectively, for predicting the clinical phenotypes of DDAs compared with the classic K-nearest neighbors method (KNN, including drug-based KNN and disease-based KNN), Random Forest, and XGBoost. By conducting leave-one-drug-class-out cross-validation, the AUC and AUPR of DDIT demonstrated an improvement of 0.135 in AUC and 0.075 in AUPR compared to any of the other four methods. Within the top 10 predicted indications, side effects, and contraindications, 7/10, 9/10, and 9/10 hit known drug–disease associations. Overall, DDIT is a useful tool for predicting multiple clinical phenotypic types of drug–disease associations.

## Introduction

Novel drug development is a complicated, time-consuming, and expensive process. It often takes 10–15 years of research and 0.8–1.5 billion dollars to bring a drug to market (Li et al., 2016). Drug repurposing provides an effective method for high-speed, low-risk drug development (Rymbai et al., 2020). One classic example is the discovery of the drug sildenafil for the treatment of male sexual dysfunction, which had been previously developed as a hypertension drug in 1989 (Ghofrani et al., 2006). Another is azidothymidine, originally failing in trials as a tumor chemotherapy drug, but then succeeding as a treatment for AIDS in 1980 (Broder, 2010). However, most of these previously successful cases of drug repositioning have relied upon individuals with a deep understanding of the pharmacology of the drug or from retrospective clinical experience, rather than from systematic or statistical analysis (Pushpakom et al., 2019).

Based on input data type, *in silico* drug repositioning is divided into four classes based on either (1) molecular structure, (2) drug–target interactions, (3) gene expression, or (4) phenotype (Duran-Frigola and Aloy, 2012).

For (1) molecular structure-based data, molecular docking is a versatile bioinformatics tool used to predict the geometry and to score the interaction of a target protein in a complex with a small-molecule drug (March-Vila et al., 2017). It requires no prior information except structural inputs from both the drug and the target and can either identify potential targets for a given drug or identify potential drugs for a specific target (Luo et al., 2016a). Liu et al., for example, developed a computational protocol named SCAR based on molecular docking to identify the possible covalent drugs targeting the main protease (3CLpro) of severe acute respiratory syndrome coronavirus 2 (*SARS* *-* *CoV* *-* *2*) (Liu et al., 2020). In addition to molecular docking, machine learning can also use structural data to make predictions. In this way, Hu et al. used convolutional neural networks to predict drug–target interactions based on drug structure and protein sequences (Hu et al., 2019); Yi et al. developed a deep gated recurrent units model to predict potential drug–disease interactions using comprehensive similarity measures and Gaussian interaction profile kernel (Yi et al., 2021); and Ke et al. established a deep neural network (DNN) to identify potential drugs for anti-coronavirus activities (Ke et al., 2020).

For (2) drug–target data, machine learning methods and network-based methods are often employed. For machine learning methods, Lu and Yu inferred unknown relationships between drugs and diseases using a regularized kernel classifier based on a unified and extended similarity kernel framework (Lu and Yu, 2018), whereas Luo et al. proposed a novel computational method named MBiRW, which utilizes some comprehensive similarity measures and a bi-random walk (BiRW) algorithm to identify potential novel indications for a given drug (Luo et al., 2016b). For network-based methods, Yu et al. also developed a computational pipeline called KDDANet for systematic and accurate uncovering of the hidden genes mediating known drug–disease associations from the perspective of a genome-wide functional gene interaction network. This utilized three existing network algorithms, namely, minimum cost network flow optimization, depth-first searching, and graph clustering (Yu et al., 2021). In addition, Zeng et al. developed a network-based deep-learning approach, termed deepDR, for *in silico* drug repurposing (Zeng et al., 2019).

For (3) gene expression data, signature mapping and machine learning are often used for drug repositioning. For signature mapping, Le et al. used a rank-based pattern matching strategy based on the Kolmogorov–Smirnov Statistic to query the signatures against drug profiles from Connectivity Map (CMap) (Lamb et al., 2006; Le et al., 2021). Wu et al. developed a database called DrugSig for computational drug repositioning utilizing gene expression signatures (Wu et al., 2017). Kim et al. used a computational reversal of gene expression to explore new drug candidates for gastric cancer (GC) (Kim et al., 2019). For machine learning, Rodriguez et al. quantified potential associations between the pathology of AD severity and molecular mechanisms to discover a list of genes associated with AD severity. Then, they apply DRIAD, a machine learning framework, to the lists of genes arising from perturbations in differentiated human neural cell cultures by 80 Food and Drug Administration (FDA)-approved and clinically tested drugs, producing a ranked list of possible repurposing candidates (Rodriguez et al., 2021).

For (4), *in silico* clinical phenotype-based screening methodologies have also provided new hypotheses to reposition drugs. Systematic analysis revealed that phenotypic screening exceeded target-based approaches in discovering first-in-class small-molecule drugs (Swinney and Anthony, 2011; Duran-Frigola and Aloy, 2012). Clinical phenotypic information comes from actual patient data that reduce the bias caused by incomplete understanding of pathogenesis and can directly help rational drug repositioning. In this way, Yang and Agrawal combined adverse effect information derived from drug labels with drug-disease relationships obtained from the PharmGKB database (Thorn et al., 2005) and were able to predict repositioning indications for 145 diseases (Yang and Agarwal, 2011). They claimed that closer attention should be paid to the side effects observed in trials, not just in evaluating the harmful effects related to the drug under trial but also in rationally exploring the repositioning potential based on this “clinical phenotypic assay.” Vogt et al. found that contraindications associated with high phenotypic similarities often involved diseases that have been reported as side effects of the drug (Vogt et al., 2014). These indicated that the known drug and clinical phenotype relationships have provided explicit repositioning hypotheses, such as drugs causing hypoglycemia are potential candidates for diabetes. However, such clinical phenotypic information has not yet been fully exploited in phenotype screening-based drug repositioning methods. To incorporate such considerations, we take into account the three types of clinical phenotype, namely, indications (Is), side effects (SEs), and contraindications (CIs), each of which are interrelated. Integrating different such phenotypic types is suggested to result in an improvement in the prediction performance for drug repositioning and help to understand drug–disease associations.

In this paper, we have compiled a multidimensional drug–disease network by systematically collecting data of clinical phenotype and proposing a restricted Boltzmann machine (RBM)-based (Hinton and Salakhutdinov, 2006) computational tool, DDIT, to predict multiple phenotypes of drug–disease associations (DDAs). The choosing of an RBM model to integrate multiple clinical phenotype data is guided by the following considerations: (1) an RBM is an energy-based two-layer graph model that can work well on a multidimensional network; (2) RBMs have been proven to have a competitive advantage in collaborative filtering (Salakhutdinov et al., 2007), drug–target interaction prediction (Wang and Zeng, 2013), and disease–microRNA association prediction (Chen et al., 2015). The primary potential use of this software is in the preclinical consideration of any potential new Is, SEs, and CIs of drugs based on existing information, thereby saving costs and providing evidence for further downstream analysis. To our knowledge, DDIT is the first computational model to simultaneously predict different phenotypes of DDAs.

## Materials and Methods

### Overview

**FIGURE 1:**
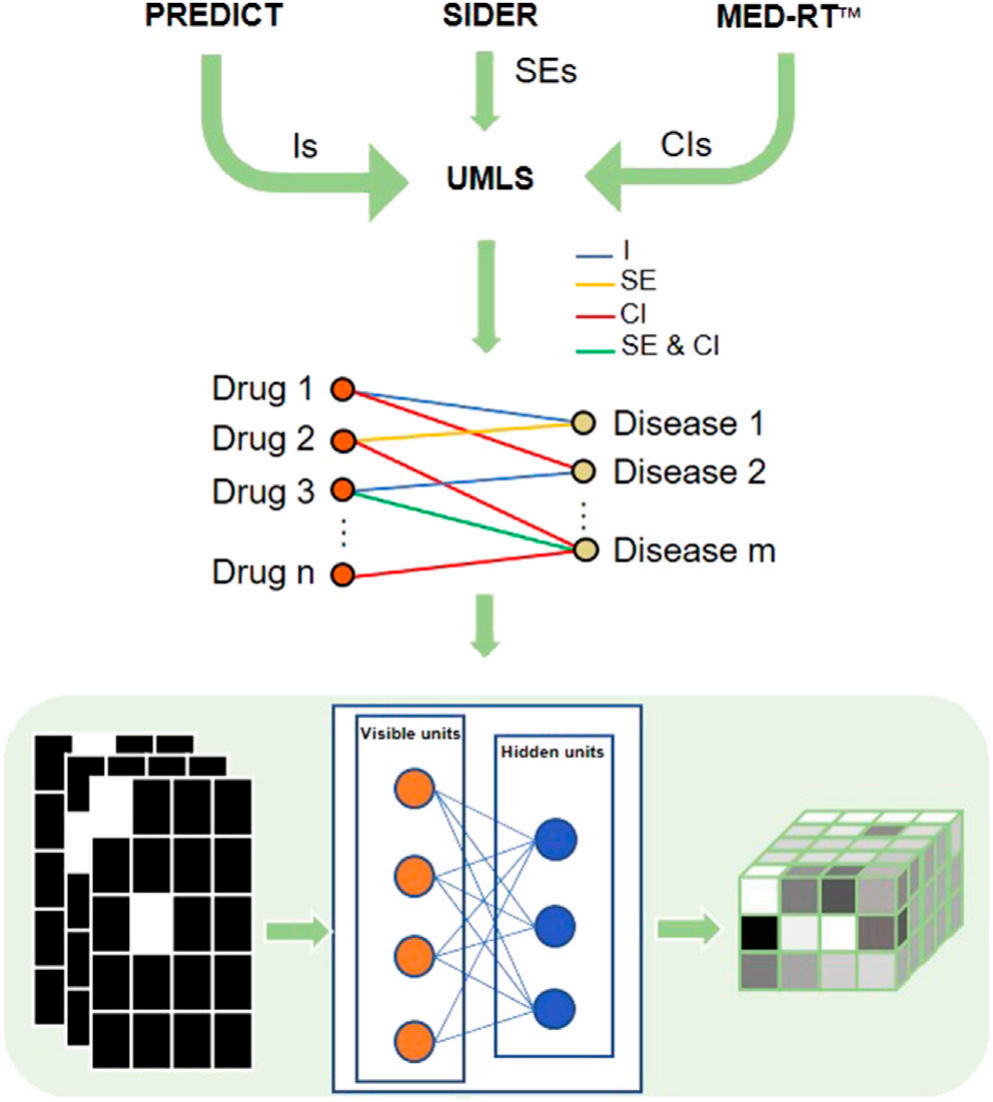
Flowchart of DDIT modeling. First, we collected different phenotypic types from PREDICT, SIDER, and MED-RT, respectively, followed by mapping the disease and drug names to UML ontologies. We then constructed a multidimensional network with nodes presenting drug or disease, and with the edge representing indication, side effect, and contraindication. The formatted data were input into a restricted Boltzmann machine (RBM) and the output reconstructed the input. We here take five drugs and four diseases as example. The left matrices represent three types of DDAs. For each matrix, the row represents drugs, while the column represents disease. The square is white if A_ij_ = 1, which means the drug and disease have indication/side effect/contraindication, black if otherwise. The right matrix is the output probability. The *y*-axis represents drug, the *x*-axis represents disease, and the *z*-axis represents the three types of DDAs. As the probability is in the range from 0 to 1, we color them in gray. I, indications; SE, side effects; CI, contraindications.

Since an RBM can be efficiently applied to learn the distribution of multidimensional networks and reconstruct their inputs, we developed an RBM-based model, DDIT, to predict different phenotypic types of DDAs. Figure 1 shows the flowchart of DDIT.

### Data Collection and Extraction

Drug indications are gold standard dataset from PREDICT (Gottlieb et al., 2011). The data for drug side effects is obtained from SIDER (Kuhn et al., 2016). The data for drug contraindications are from MED-RT (https://ncit.nci.nih.gov/ncitbrowser/pages/vocabulary.jsf?dictionary=MED-RT), produced by The Veterans Health Administration (VHA). For this, we downloaded the archive content Core_MEDRT_2019.11.04_XML.zip (https://evs.nci.nih.gov/ftp1/MED-RT/Archive/) and then extracted the relationship of “CI-with,” which describes co-morbid contraindication of a drug (see Supplementary Figure S1). In total, we collected 2,816 drug–indication pairs, 132,150 drug–SE pairs, and 10,443 drug–contraindication pairs.

### Data Mapping

As the names of drugs and diseases in different datasets often vary in their vocabulary, this required consideration and adjustment for standardization. For example, the drug names in the indication dataset, side effect dataset, and contraindication dataset were from DrugBank (Wishart et al., 2007), ATC (Miller and Britt, 1995), and RxNorm (Nelson et al., 2011), respectively, while the corresponding disease names in these three datasets were from OMIM (Hamosh et al., 2005), UMLS (Bodenreider, 2004), and MeSH (Leydesdorff et al., 2016), respectively. To unify the drug and disease names, we mapped their names to Unified Medical Language System (UMLS) ontology. We accessed UMLS Knowledge Sources Metathesaurus 2019AB using Rest API for Java (https://github.com/HHS/uts-rest-api). All the data were completely mapped to UMLS ontology, and no other tools were used to unify these drug and disease terms.

### Data Balance

During the data collection phase, we collected 2,816 drug–indication pairs, 132,150 drug–SE pairs, and 10,443 drug–contraindication pairs. As these data sets were unbalanced, to balance the data, we first selected a disease subset *S*, then we randomly select 2,816 (2,816 is the lowest count among three types of DDAs) associations from drug–SEs and 2,816 associations from drug–contraindications with disease in the subset *S*. To obtain *S*, we have selected the diseases meeting to one of following two criteria: (1) diseases included in the known drug indications and (2) diseases shared by two different types of clinical phenotypes. Finally, we obtained the 2,816 data points for each type of associations.

### The RBM Model

**FIGURE 2:**
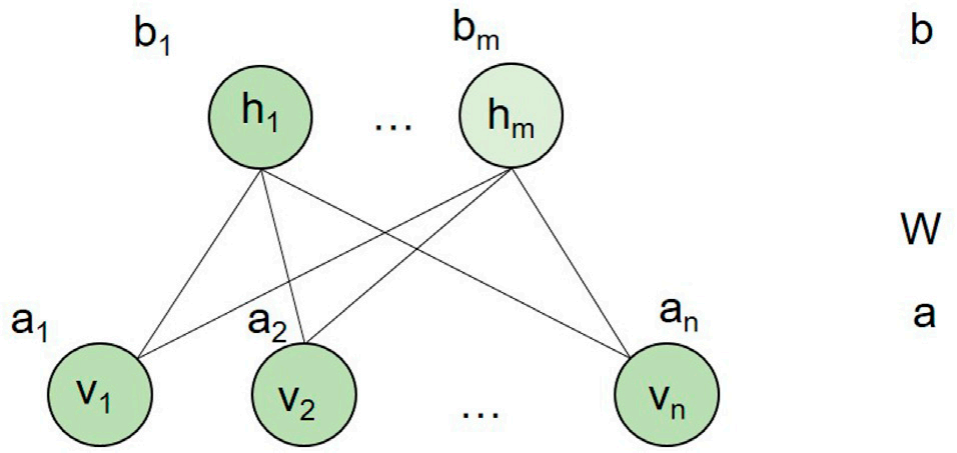
An RBM model. *n* and *m* are the number of visible units and hidden units, respectively. ***a*** is the bias of visible variables, while ***b*** is the bias of hidden variables. ***W*** is the connection weight matrix between each visible unit and each hidden unit.

An RBM is an undirected graphic model (Salakhutdinov et al., 2007) that can be used to learn probability distributions over input data using a layer of binary hidden units. As shown in Figure 2, an RBM consists of a layer of visible units (*v*) and a layer of hidden units (*h*). Each visible unit is connected to all hidden units and has no intralayer connections between any pairs of visible units or any pairs of hidden units. The state of each unit possesses a binary value.

**FIGURE 3:**
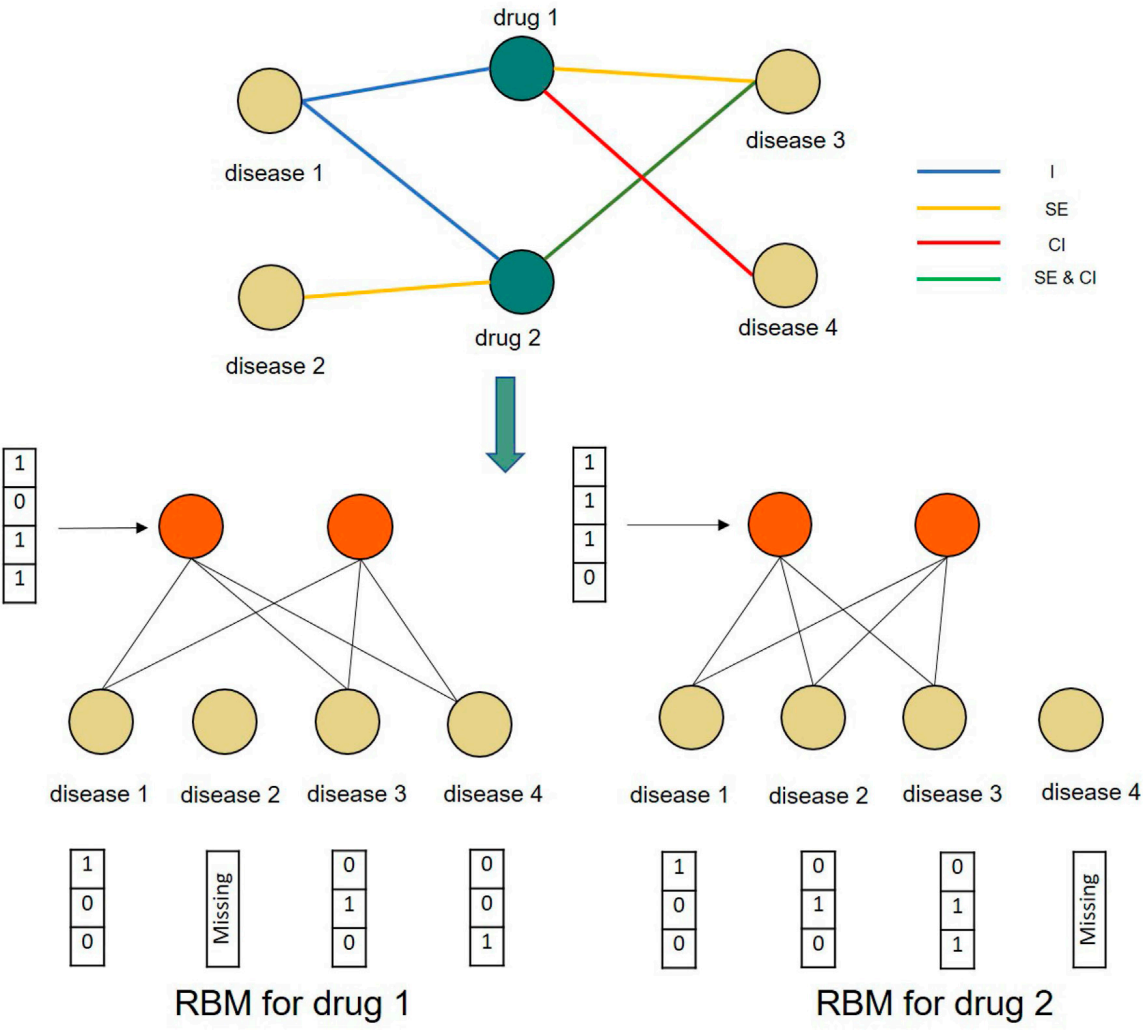
A toy example of constructing conditional RBMs for two drugs and four diseases. The RBMs for both drug 1 and drug 2 share the same parameters.

In our study, we built an RBM model for each drug. In other words, for a drug, we adopted a two-layer RBM with diseases as visible layer and 400 hidden units as hidden layer. The hidden layer represented the hidden factor, and it cannot be observed. Each RBM model for a drug only had diseases related to the drug as visible units. Thus, different drugs had different RBM models. However, different RBMs of drugs shared the connection weight between each visible disease unit and hidden unit pairs. We assumed that for each drug, the RBM model had *n* visible units, m hidden units, and *l* association types encoded in a visible unit. In our context, each visible unit represented a disease. Therefore, we let binary vector $v_{i}=\left(v_{i}^{1},\cdots ,v_{i}^{k},\cdots ,v_{i}^{l}\right)$ denote the state of the *i*th visible unit, where visible variables $v_{i}^{k}=1$ if the *k*th type of DDA is observed in the input data, and $v_{i}^{k}=0$ otherwise. For example, for indication, the binary vector is ***v***_***i***_ = (1,0,0), and for both side effect and contraindication, the binary vector is ***v***_***i***_ = (0,1,1). With a 3-bit vector, it will be able to distinguish the three types of DDA at the same time. For each hidden unit, the state of *jth* hidden unit is expressed as $h_{j},\;j=1,2,\cdots ,m.$ Let $W_{ij}^{k}$ denote the weight of the connection between visible variable $v_{i}^{k}$ and hidden variable $h_{j}$ , and it is shared by different RBMs of drugs*.* The vector $v=\left(v_{1},v_{2},\cdots ,v_{n}\right)$ denotes the input layer, while ***h*** $=\left(h_{1},h_{2},\cdots ,h_{m}\right)$ denotes the hidden layer. Figure 3 shows the modeling of four drugs and two diseases. Through the CD algorithm, the model can be effectively trained (Hinton, 2002). An RBM can learn the distribution of multidimensional networks well and reconstruct the input. This will predict the DDAs that are not yet known in the input. RBM details are supplied as Supplementary Material (Supplementary Text S1).

As the verified drug–disease associations provide more reliable information than those that are as yet unknown, we further introduced a conditional RBM to incorporate this additional information to affect the states of hidden units (Salakhutdinov et al., 2007). In this, we let $r_{i}=\left(r_{1},\cdots ,r_{j},\cdots ,r_{m}\right)$ be a binary vector, in which $r_{i}=1$ if disease j has association with the current drug i, and $r_{i}=0$ otherwise. Details about the conditional RBMs are supplied as Supplementary Material (Supplementary Text S2).

The algorithm is implemented in Python. Through grid search, we determined the best parameters: (“m”: 400, “learning_rate”: 0.5, “epochs”: 300).

### K-Nearest Neighbors

According to the computational method of similarity, KNN (Guo et al., 2003) was divided into drug- and disease-based KNN. For drug-based KNN, the hypothesis was that similar drugs should have similar effects on the same disease. For any given drug, we identified the other top *k* drugs that were most similar to it and then calculated its phenotypes by averaging the phenotypes of its neighbor drugs. The drug similarity is calculated by Jaccard Index based on the drug-related disease profile with the formula defined in Eq. 1 as follows:

$$J=\frac{M_{11}}{M_{01}+M_{10}+M_{11}}$$ (1)

The approach for disease- and drug-based KNN was similar where the similarity between two diseases was calculated by disease-related drug profile similarity.

For KNN, we further optimized for *k* systematically. For drug-based KNN, we searched *k* ranging from (1, 10) and choose a *k* = 6 for the best AUC and AUPR with 0.736 and 0.824, respectively, in the indication prediction. For disease-based KNN, we searched *k* ranging from (1, 10) and choose *k* = 5 for the best AUC and AUPR with 0.777 and 0.916, respectively, in indication prediction.

### Random Forest

We adopted Random Forest as a classifier for comparative analysis. For each drug–disease pair, we combined the drug-related disease profile and disease-related drug profile together as the feature vector to train the Random Forest prediction model. For three association types, we constructed three random forest models, respectively. Each model was a problem of binary classification. We implemented this algorithm by using the “RandomForestClassifier” function in the sklearn package with default parameters.

### XGBoost

We further carried out XGBoost on our dataset. We modeled the same data as the input of random forest. We adopted the Scikit–Learn Wrapper interface for XGBoost to create the XGBoost model. We optimized the hyper parameters using “GridSearchCV” function and obtained the best parameters (“gamma”: 0.25, “learning_rate”: 0.1, “max_depth”: 5, “reg_lamba”: 0, “scale_pos_weight”: 1). We then trained the model with the best parameters.

### Tenfold Cross-Validation

We used the 10-fold cross-validation method to evaluate the model. In this method, the data set was randomly divided into 10 sub-parts with nine of them used as the training set in turn and the remaining one being the test set.

### Leave-One-Drug-Class-Out Cross-Validation

To access how trained models can be generalized into groups of drugs that the models have never trained on before, we further make a leave-one-drug-class-out cross-validation (Yao et al., 2019). We first mapped the UMLS concept to the ATC code, then divided the drugs into 15 classes by the ATC code (See Supplementary Table S1). The data sets were divided into 15 parts according to drug classes. Fourteen of them were used as the training set in turn, the remaining one being the test set.

### Leave-One-Disease-Class-Out Cross-Validation

To access how trained models can be generalized into groups of diseases that the models have never trained on before, we further made a leave-one-disease-class-out cross-validation. We divided the diseases into 23 MSH classes (see Supplementary Table S2). Twenty-two of them were used as the training set in turn, the remaining one being the test set.

### Web Server

The web server of DDIT was built using modern frontend–backend architecture with three main components: front end, backend server, and a relational database containing the information of drugs, diseases, and their phenotypic associations. The database was built using Mysql 5.6. The backend was implemented in Java using SSM (Spring + SpringMVC + Mybatis) as a framework and provided the REST API (Sohan et al., 2017). The front-end was built using React (Gackenheimer, 2015) and several other libraries. The backend was deployed in Apache Tomcat (Vukotic and Goodwill, 2011), while the front-end was deployed in Nginx (Nedelcu, 2013). This architecture provided for the easy maintenance of each module.

## Results

### DDIT Performance

**FIGURE 4:**
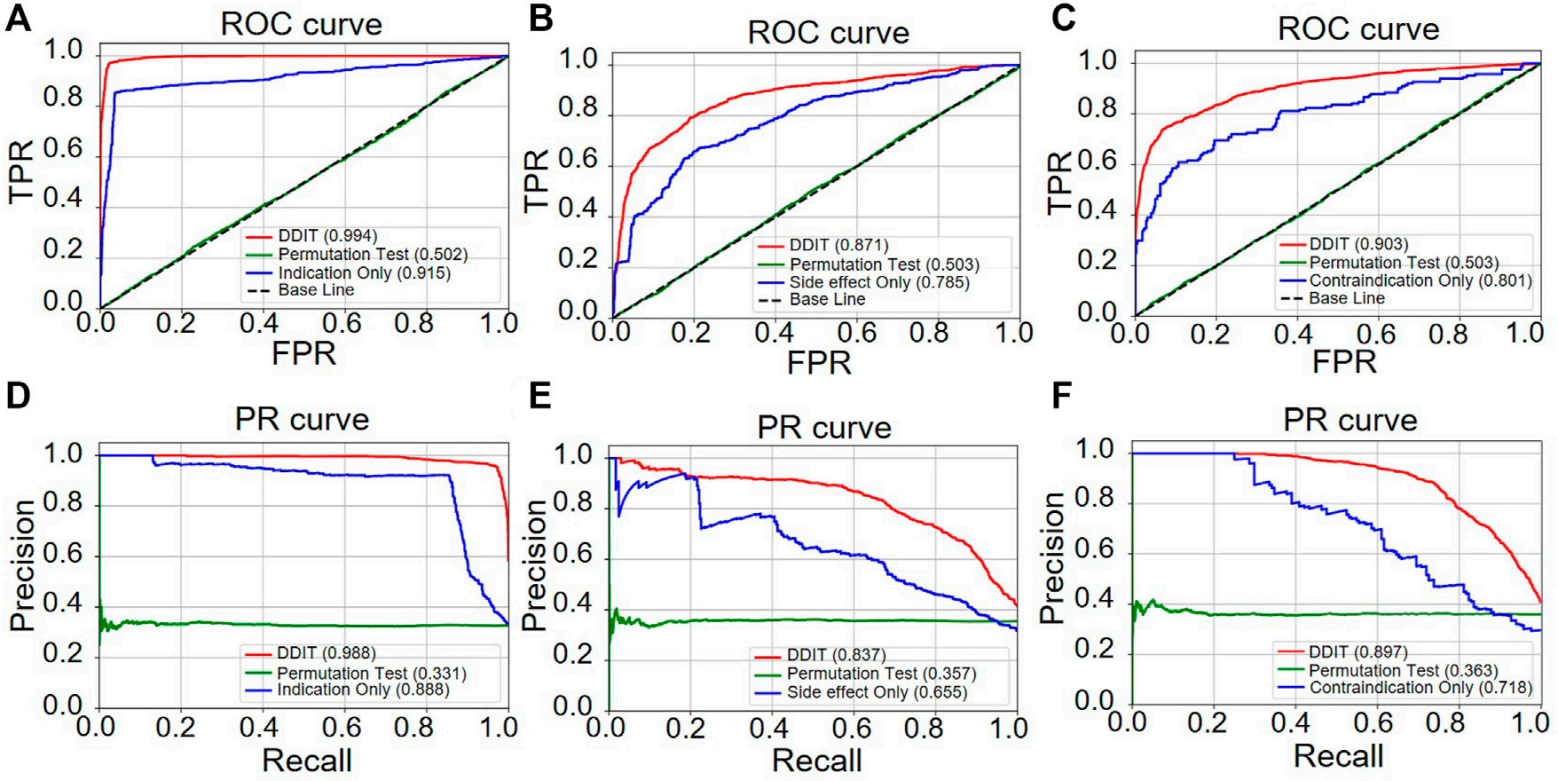
ROC and PR curve of DDIT compared with permutation test and single phenotypic type data only. **(A,D)** ROC and PR curves comparing DDIT with permutation tests and indication data only for indication predictions of known drugs. **(B,E)** ROC and PR curves comparing DDIT with permutation test and side effect data only for side effect predictions of known drugs. **(C,F)** ROC and PR curves comparing DDIT with permutation test and contraindication data only for contraindication predictions of known drugs.

Receiver operator characteristic (ROC) and precision–recall (PR) curves were used as evaluation metric for predictive performance. We compared DDIT integrating three phenotypic types of DDAs with one single phenotypic type. Both in terms of ROC (Figure 4A) and PR curves (Figure 4D), DDIT with integrated three phenotypic types performed >0.079 better than single indication data in indication prediction. Similarly, in prediction of side effect and contraindication, area under ROC curve (AUC) and area under the PR curve (AUPR) had improved by >0.086 (Figures 4B, E) and >0.102 (Figures 4C,F) respectively. This suggests that data integrating multiple clinical phenotypic types provide more information than single analysis and simultaneously improve prediction performance.

### Comparison With Other Methods

**FIGURE 5:**
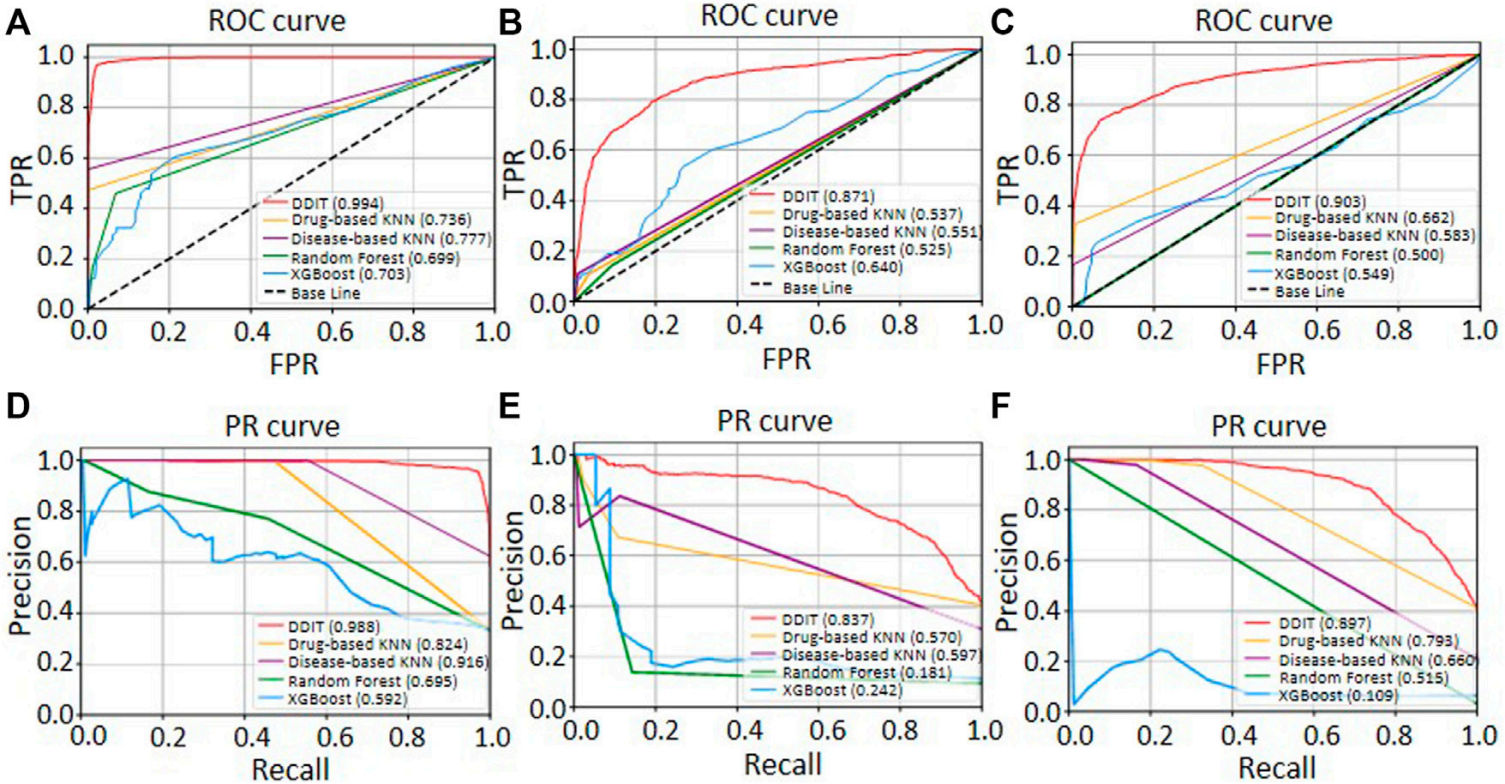
ROC and PR curves of DDIT compared with drug-based KNN, disease-based KNN, Random Forest classifier, and XGBoost. **(A,D)** ROC and PR curves comparing DDIT with drug-based KNN, disease-based KNN, Random Forest, and XGBoost for novel indication prediction of known drugs. **(B,E)** ROC and PR curves comparing DDIT with drug-based KNN, disease-based KNN, Random Forest, and XGBoost for novel side effect prediction of known drugs. **(C,F)** ROC and PR curves comparing DDIT with drug-based KNN, disease-based KNN, Random Forest, and XGBoost for novel contraindication prediction of known drugs.

We then evaluated the performance by comparing DDIT with the drug-based KNN, disease-based KNN, Random Forest, and XGBoost (see *Materials and Methods*). As shown in Figure 5, DDIT represented improvement by at least 0.217 in AUC and 0.072 in AUPR compared with the other four methods. The AUC and AUPR for leave-one-drug-class-out is also shown in Supplementary Figure S2. Here, DDIT represented improvement of at least 0.135 in AUC and 0.075 in AUPR, compared to the other four methods.

### The Applications of DDIT to Multiple Clinical Phenotypic Types

We then searched for an external validation dataset from CTD (Davis et al., 2021), DrugBank (Davis et al., 2021), and DynaMed (https://www.dynamed.com/) to evaluate the prediction results. We collected the novel DDAs from the CTD database. These had not been used for building DDIT, but the drugs and diseases of these novel DDAs were contained in our modeling datasets. Table 1 shows the top 10 predicted indications. Seven of these 10 predictions could be found in CTD, DrugBank, or DynaMed databases. The remaining three predictions may represent candidate drugs for new indications. For example, DDIT predicted that Bleomycin is indicated for small cell lung cancer. This is conceivable as a true positive, since Bleomycin, as recorded in DrugBank, is a drug for the treatment of malignant neoplasms and operates by inhibiting DNA synthesis. That Buspirone is a candidate drug for obsessive–compulsive disorder may also be true positive, as it is labeled as indicated for anti-anxiety in DynaMed, which is a symptom of obsessive–compulsive disorder.

**TABLE 1:** Top 10 scoring indications by DDIT.

Drug CUI	Drug name	Disease CUI	Disease name	Evidence
C0392938	Zoledronate	C0029459	Osteoporosis, senile	CTD
C0392938	Zoledronate	C0029458	Osteoporosis, postmenopausal	DrugBank
C0014912	Estradiol	C4722327	Prostate cancer, hereditary, 1	DynaMed
C0020823	Ifosfamide	C0149925	Small cell carcinoma of lung	CTD
C0030899	Pentoxifylline	C1858361	Pyogenic arthritis, pyoderma gangrenosum and acne	—
C0004147	Atenolol	C1837014	Atrial fibrillation, familial, 3	DrugBank
C0059985	Fludarabine	C0023467	Leukemia, myelocytic, acute	CTD
C0005740	Bleomycin	C0149925	Small cell carcinoma of lung	—
C0123091	Quetiapine	C0036341	Schizophrenia	CTD
C0006462	Buspirone	C0028768	Obsessive–compulsive disorder	—

For the prediction of side effect, Table 2 shows the top 10 predicted side effects. Nine of 10 can be found in DynaMed. DDIT inferred that dermatitis was a side effect of vincristine. Vincristine is a chemotherapy medication used to treat various types of cancer. The prevalent cutaneous side effects in patients affected by tumors undergoing chemotherapy are skin rash, xerosis, pruritus, paronychia, hair abnormality, and mucositis (Fabbrocini et al., 2012). This may suggest that our inference is again a possible true positive.

**TABLE 2:** Top 10 scoring side effects by DDIT.

Drug CUI	Drug name	Disease CUI	Disease name	Evidence
C0042523	Verapamil	C0018681	Headache	DynaMed
C0031469	Phenylephrine	C0027497	Nausea	DynaMed
C0016365	Fluoxetine	C0042963	Vomiting	DynaMed
C0008809	Ciprofloxacin	C0027497	Nausea	DynaMed
C0073571	Ropivacaine	C0027497	Nausea	DynaMed
C0529793	Sildenafil	C0017178	Gastrointestinal Diseases	DynaMed
C0216784	Valsartan	C0018681	Headache	DynaMed
C0529793	Sildenafil	C0035455	Rhinitis	DynaMed
C0035608	Vincristine	C0011603	Dermatitis	—
C0021246	Indomethacin	C0011991	Diarrhea	DynaMed

As for contraindications, Table 3 shows the top 10 predicted contraindications. Nine of ten could be found in DynaMed. The prediction atrioventricular block as a contraindication of atenolol may also be true positive as DynaMed notes that atenolol can cause atrioventricular blocks in cases of severe positioning.

**TABLE 3:** Top 10 scoring contraindications by DDIT.

Drug CUI	Drug name	Disease CUI	Disease name	Evidence
C0033497	Propranolol	C0036980	Shock, cardiogenic	DynaMed
C0076840	Torsemide	C0003460	Anuria	DynaMed
C0027302	Nadolol	C0428977	Bradycardia	DynaMed
C0015011	Ethinyl estradiol	C0034065	Pulmonary embolism	DynaMed
C0025598	Metformin	C0011880	Diabetic ketoacidosis	DynaMed
C0289313	Rosiglitazone	C0011880	Diabetic ketoacidosis	DynaMed
C0002598	Amiodarone	C0037052	Sick sinus syndrome	DynaMed
C0072857	Quinapril	C0020649	Hypotension	DynaMed
C0004147	Atenolol	C0004245	Atrioventricular block	–
C0028356	Norethindrone	C1458155	Mammary neoplasms	DynaMed

Altogether, these results suggest that DDIT is a powerful computational tool that integrates multiple clinical features for the facilitation of drug repurposing.

### Web Interface

**FIGURE 6:**
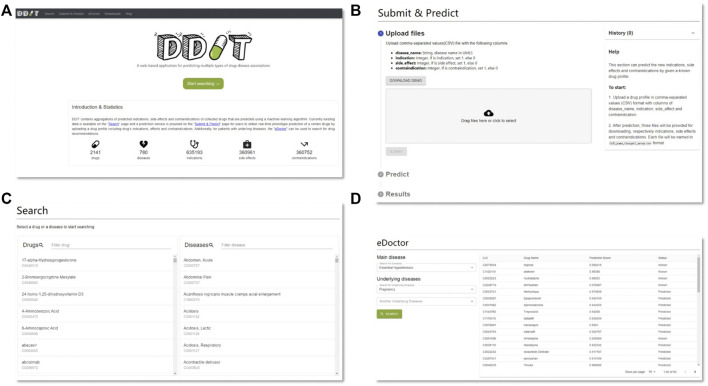
DDIT web interface. **(A)** Home page. **(B)** Prediction page. Predicting clinical phenotypic types based on drug profiles submitted by users. **(C)** Search page. Left: search drug’s Is, SEs, and CIs by drug’s name; right: search for drugs that can cure, cause the disease, or as contraindicated in people with the disease by disease’s name. **(D)** eDoctor page. Provide recommended drugs for patients with underlying diseases.

Figure 6 represents the web interface of DDIT. Three core functions are implemented in DDIT:

– Drug/Disease: The page allows users to search for either (i) predicted Is, SEs, and CIs by inputting a drug name or (ii) predicted related that can cure, cause the disease, or as contraindicated in people with the disease, by giving the disease name.
– Submit and Predict: The page executes real-time phenotype prediction of DDAs based on drug profiles, including drug Is, SEs, and Cis, as submitted by users.
– eDoctor: This page provides recommended drugs for patients with underlying diseases.

### Data Collection

The exact data for the RBM is a 3D array (2141*780*3) named ***A*** in .npy format in Numpy (provided in Supp. Data S1 file). The 0^th^ dimension represents drugs, ***A*** *[i,,]* means the *i-th* drug; the 1^th^ dimension represents diseases, ***A*** *[,j,]* means the *j-th* disease; the 2^th^ dimension represents the phenotypic types. ***A*** *[i,j,k]* means the *k-th* type between drug *i* and disease *j*. For example, ***A*** *[i,j,0]* denotes indication between drug *i* and disease *j*, ***A*** *[i,j,1]* denotes side effect between drug *i* and disease *j*, while ***A*** *[i,j,2]* denotes contraindication between drug *i* and disease *j*. The index *i* and *j* is calculated by the drug_id -1 and disease_id -1 respectively because the array in numpy starts from index 0 rather than 1. The mapping between drug id and drug name is provided in Supp. Data S2 file, while the mapping between disease id and disease name is provided in Supp. Data S3 file.

The ranked predictions of three types are provided in supplementary files of Supp. Data S4, Supp. Data S5, Supp. Data S6 respectively. The first column represents drug id, the second column is disease id, the third column is the prediction score, and the last column represents the status, status = 0 if the association type is not included in our dataset, status = 1 if the association type is known in our dataset.

## Discussion

DDIT is a user-friendly web server that facilitates researchers to explore potential clinical phenotypes of DDAs. The main contributions are as follows: (i) simultaneous prediction of multiple phenotypes of DDAs based on the integration from distinct datasets with respective clinical phenotypes; (ii) prediction of real-time potential phenotypes of a drug of interest, including Is, SEs, and CIs, by uploading drug profiles; and (iii) preliminary drug screening for patients with underlying diseases. One shortcoming is represented in that our study observed that an RBM cannot make predictions for a disease class without any known related drugs (AUC, ∼0.549). That is because, in our model, we view each visible unit as a disease, and the model then learns the similarity of different diseases. As for a site that recommends movies to watch would find it difficult to process a recommendation for a movie that nobody has ever seen or reviewed, it would be hard for this model to predict drugs for a new disease class that had no prior drug associations. To validate this, we further used drugs as visible units and built RBM for each disease. We want to see if it can make good prediction for leave-one-disease-class-out. As expected, the AUC and AUPR is 0.822 and 0.803 for indication, 0.770 and 0.689 for side effect, and 0.876 and 0.795 for contraindication, respectively. These results have further demonstrated that, using drug as visible unit, RBM can capture the similarity of different drugs and can make good prediction for leave-one-disease-class-out. In the future, we will expand the number of drugs, diseases, and their associations, and integrate this knowledge into DDIT for further aiding drug repositioning. We will also try to collect more data and use DDIT to reposition drugs for COVID-19. We believe that our work will provide an additional layer, providing positive contributions towards drug repositioning.

## Data Availability

Publicly available datasets were analyzed in this study. Indications were from paper (https://doi.org/10.1038/msb.2011.26), side effects were from SIDER (http://sideeffects.embl.de/), and contraindications were from MED-RT (https://ncit.nci.nih.gov/ncitbrowser/pages/vocabulary.jsf?dictionary=MED-RT).

## Abbreviations

AUC, area under ROC curve; AUPR, area under PR curve; CIs, contraindications; DDAs, drug–disease associations; Is, indications; PR, precision–recall; ROC, receiver operator characteristic; RBM, restricted Boltzmann machine; SEs, side effects.
