# Supplementary material for: DDIT: An Online Predictor for Multiple Clinical Phenotypic Drug-Disease Associations
Source: Front Pharmacol. 2022 Jan 19;12:772026. doi: 10.3389/fphar.2021.772026 (PMC8809407; doi:10.3389/fphar.2021.772026)
Supplement: Supplementary file 1 [file DataSheet1.ZIP › supplementary/Supp.supplementary material 2021.11.14.docx]

**Supplementary Materials** **for “****DDIT:** **an online predictor for** **multiple clinical phenotypic types of drug-disease associations”**

**Legends of Supplementary Figures, Table and Texts:**

**Figure S1.** Dataflow diagram of extracting drug-contraindication associations from MED-RT.

**Figure S2.** The AUC and AUPR of different types of machine learning methods in leave-one-drug-class-out evaluation.

**Table S1.** Drug classification according to their ATC codes.

**Table S2.** Disease classification according to their MSH codes.

**Text S1.** RBM model

**Text S2.** Conditional RBM

**
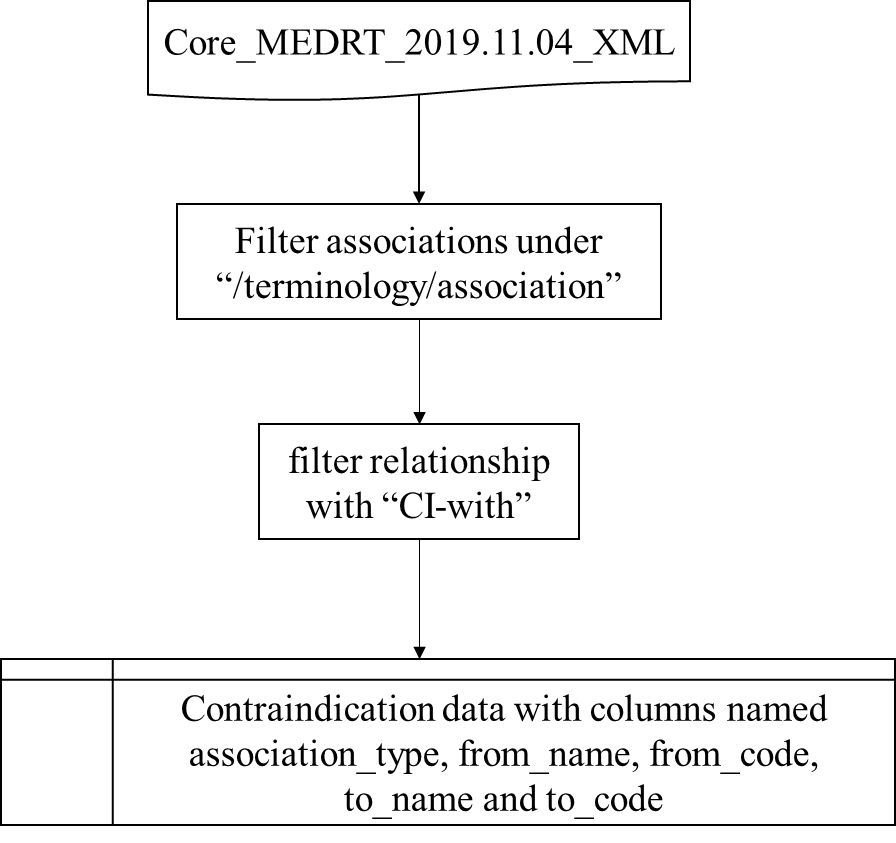
**

**Figure S1. Dataflow diagram of extracting drug-contraindication associations from MED-RT.** we first extract the association nodes under “/terminology/association” using SAX in Java, then we import all the associations to Mysql database, and filter relationship with “CI-with”. The processed data has 5 columns named association_type, from_name, from_code, to_name and to_code respectively. The association_type represents the type between two drug and disease, here is “CI-with” which means “contraindication”; from_name means the drug name in MeSH namespace; from_code means the drug id in MeSH namespace; to_name means disease name in RxNorm namespace and to_code means disease id in RxNorm. The relationship between drug and disease has a qualifier from MED-RT.


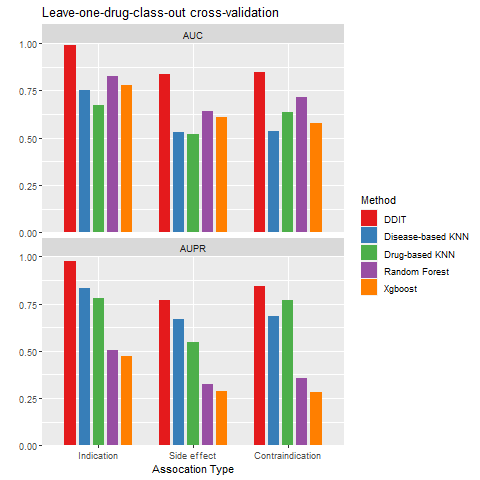


**Figure S2. The AUC and AUPR of different types of machine learning methods in leave-one-drug-class-out evaluation.**

| **Drug class** | **Drug number** |
| --- | --- |
| Alimentary tract and metabolism | 124 |
| Antiinfectives for systemic use | 124 |
| Antineoplastic and immunomodulating agents | 137 |
| Antiparasitic products, insecticides, and repellents | 18 |
| Blood and blood forming organs | 71 |
| Cardiovascular system | 149 |
| Dermatologicals | 45 |
| Genito urinary system and sex hormones | 65 |
| Musculo-skeletal system | 58 |
| Nervous system | 216 |
| Respiratory system | 68 |
| Sensory organs | 71 |
| Systemic hormonal preparations, excl. sex hormones and insulins | 32 |
| Various | 54 |
| Unclassfied | 909 |

## Table S1. Drug classification according to their ATC codes

**Table S2. Disease classification according to their MSH codes**

| **Disease class** | **Disease number** |
| --- | --- |
| Behavior and Behavior Mechanisms | 3 |
| Cardiovascular Diseases | 40 |
| Chemically-Induced Disorders | 3 |
| Congenital, Hereditary, and Neonatal Diseases and Abnormalities | 71 |
| Digestive System Diseases | 42 |
| Endocrine System Diseases | 12 |
| Eye Diseases | 18 |
| Female Urogenital Diseases and Pregnancy Complications | 26 |
| Hemic and Lymphatic Diseases | 21 |
| Immune System Diseases | 13 |
| Infections | 17 |
| Male Urogenital Diseases | 5 |
| Mental Disorders | 5 |
| Musculoskeletal Diseases | 13 |
| Neoplasms | 56 |
| Nervous System Diseases | 30 |
| Nutritional and Metabolic Diseases | 39 |
| Otorhinolaryngologic Diseases | 3 |
| Pathological Conditions, Signs and Symptoms | 116 |
| Respiratory Tract Diseases | 22 |
| Skin and Connective Tissue Diseases | 18 |
| Stomatognathic Diseases | 3 |
| Unclassified | 204 |

## Text S1. RBM model

In our RBM model, the clinical phenotypic types of drug-disease associations correspond to “visible" units of the RBM because their states are observed; the feature detectors correspond to “hidden" units. We encoded the observed phenotypes of drug-disease associations using binary vectors. We encoded indication, side effect and contraindication using binary vector (1,0,0), (0,1,0) and (0,0,1) respectively. If an interaction was both side effect and contraindication, it was encoded with (0, 1, 1). The binary states of visible variables for those missing drug-disease associations were treated as missing value. To dealing with missing values, we pretended they do not exist by using RBMs with different numbers of visible units for different training cases. The different RBMs formed a family of different models with shared weights.

To obtain the distribution probability over input data, we first define the energy of a RBM system in a scalar form:

Where , are the number of visible units, hidden units and is the number of phenotypic types in a visible unit. As we investigated three phenotypic types (indication, side effect and contraindication) between drug and diseases, we set to 3.  denotes the state of the i-th visible unit, where if the k-th phenotypic type is observed in input data, and otherwise. is the state of j-th hidden unit. and are responding the bias of visible units and hidden units. is the weight matrix between each visible variable and each hidden variable .

For vector representation, we let *v* = (*v1, v2, …, vn*) and *h* = (*h1, h2, …, hm*). According to the laws of thermodynamics, the probability distribution of state (v, h) is defined as:

(2)

Where is normalized factor, which is also called partition function. It makes sum of *p*(*v,h*) be equal to 1. Combining Equation (1) and Equation (2), we can find that high energy associates with low probability, vice versa.

For our problem, the probability distribution of visible data , which is also the likelihood function, corresponds to the marginal distribution of :

(3)

Here, we also can obtain the conditional probability of h over v. The conditional Bernoulli distribution for hidden features is defined as:

(4)

Where σ is the logistic function, it implies the hidden unit will turn on when it exceeds a certain threshold. The state of each hidden unit comes from visible units and is independent with other hidden units, so we can compute the probability all in parallel.

Similarly, the conditional probability of v over h is also a Bernoulli distribution:

(5)

The conditional probability of h over v and v over h will be used in the preceding training process.

## Train

The goal of training RBM is to maximize the likelihood function, or in other word, to minimize the energy. The likelihood function is described as bellow:

By gradient ascent algorithm, we can update the parameters:

(6)

(7)

(8)

Where  is the learning rate. is the positive phase which computes the average value of for all pairs of a visible and a hidden unit. It is easy to calculate from the input data. is the negative phase which computes the average value of from the model data [1]. Exact computing is tractable, so we use the sampling method to calculate it. For example, Gibbs sampler [2], initialized at the data, for T full steps. However, it is costing as each time we update parameters, we do Gibbs sampling. Hinton, et al introduced a shortcut described as CD-k algorithm [3]. Instead of using the statistics measured in T steps, we used the statistics measured after doing one update of the Gibbs sampling. This starts by setting the states of the visible units to a training vector. Then the binary states of the hidden units are all computed in parallel using Equation (4). Once binary states have been chosen for the hidden units, update all the visible units in parallel to get a reconstruction using Equation (5). Then update the hidden units again. This is not following the gradient of the log likelihood, but it works well. The parameters update process using CD-k algorithm is as follows:

(9)

(10)

(11)

Here, represents the expectation of a distribution of samples from running the Gibbs sampling (Equation (4-5)) for T full steps. Here, we set T to 1.

## Prediction

The probability with each hidden unit will turn on is show in Equation (12). It gets from the visible units and quite independent of other hidden units, so we can compute the probability all in parallel. The reconstruction of the input data is calculated in Equation (13) [4]:

(12)

(13)

Where is the conditional probability of hidden variable given visible data .

## Text S2. Conditional RBM

As verified drug-disease associations provide more reliable information than those unknown, we introduced conditional RBM to incorporate this additional information [5] to affect the states of hidden units. Let be a binary vector of length M (total number of diseases), indicating which disease has association with the current drug (show in Figure 3). In a condition RBM, a vector will affect the states of hidden units:

(14)

Where  is a parameter describing the effect of on . The parameter can also been learned using CD algorithm:

(15)

## Reference

[1] I. Goodfellow, Y. Bengio, and A. Courville, Deep learning, MIT press, 2016.

[2] A.E. Gelfand, Gibbs Sampling. Journal of the American Statistical Association 95 (2000) 1300-1304.

[3] G.E. Hinton, Training products of experts by minimizing contrastive divergence. 14 (2002) 1771–1800.

[4] Y. Wang, and J. Zeng, Predicting drug-target interactions using restricted Boltzmann machines. Bioinformatics 29 (2013) i126-34.

[5] R. Salakhutdinov, A. Mnih, and G. Hinton, Restricted Boltzmann machines for collaborative filtering, Proceedings of the 24th international conference on Machine learning, Association for Computing Machinery, Corvalis, Oregon, USA, 2007, pp. 791–798.
